# Diurnal expression of *Dgat2* induced by time‐restricted feeding maintains cardiac health in the *Drosophila* model of circadian disruption

**DOI:** 10.1111/acel.14169

**Published:** 2024-04-14

**Authors:** Yiming Guo, Farah Abou Daya, Hiep Dinh Le, Satchidananda Panda, Girish C. Melkani

**Affiliations:** ^1^ Department of Pathology, Division of Molecular and Cellular Pathology Heersink School of Medicine, University of Alabama at Birmingham Birmingham Alabama USA; ^2^ Regulatory Biology Laboratory Salk Institute for Biological Studies La Jolla California USA

**Keywords:** cardiac health, circadian disruption, *Dgat2*, time‐restricted feeding, transcriptome analysis, triglyceride metabolism

## Abstract

Circadian disruption is associated with an increased risk of cardiometabolic disorders and cardiac diseases. Time‐restricted feeding/eating (TRF/TRE), restricting food intake within a consistent window of the day, has shown improvements in heart function from flies and mice to humans. However, whether and how TRF still conveys cardiac benefits in the context of circadian disruption remains unclear. Here, we demonstrate that TRF sustains cardiac performance, myofibrillar organization, and regulates cardiac lipid accumulation in *Drosophila* when the circadian rhythm is disrupted by constant light. TRF induces oscillations in the expression of genes associated with triglyceride metabolism. In particular, TRF induces diurnal expression of diacylglycerol O‐acyltransferase 2 (*Dgat2*), peaking during the feeding period. Heart‐specific manipulation of *Dgat2* modulates cardiac function and lipid droplet accumulation. Strikingly, heart‐specific overexpression of human *Dgat2* at ZT 0–10 significantly improves cardiac performance in flies exposed to constant light. We have demonstrated that TRF effectively attenuates cardiac decline induced by circadian disruption. Moreover, our data suggests that diurnal expression of *Dgat2* induced by TRF is beneficial for heart health under circadian disruption. Overall, our findings have underscored the relevance of TRF in preserving heart health under circadian disruptions and provided potential targets, such as *Dgat2*, and strategies for therapeutic interventions in mitigating cardiac aging, metabolic disorders, and cardiac diseases in humans.

## Graphical abstract

TRF mitigates aging and circadian rhythm disruption cardiac dysfunction including maintaining myofibrillar organization and lipid metabolism. TRF induces the expression rhythmicity of genes linked with triglyceride metabolism and induces diurnal expression of *Dgat2* with peak expression during the feeding phase under circadian rhythm disruption. Diurnal expression Overexpression of hDgat2 improves cardiac function under circadian disruption.

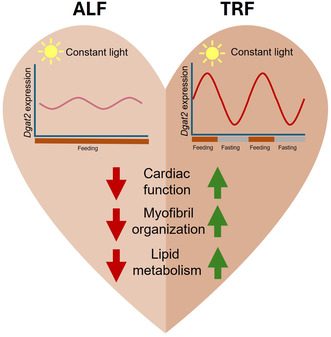

**Abbreviations**
ALF: ad‐libitum feeding
ATGL: Adipose triglyceride lipase
bmm: brummer
Dgat2: diacylglycerol O‐acyltransferase 2
LD group: 12 h light: 12 h dark cycle
LS group: constant light
TRE: time‐restricted eating
TRF: time‐restricted feeding
ZT: zitgeber time

## 1 INTRODUCTION

About 16% of the U.S. workforce works outside of a conventional daytime schedule (7 AM–6 PM) (STATISTICS USBOL, 2019), including evening shift, night shift, rotating shift, and split shift, and thus are considered shift workers. Shift workers frequently experience circadian disruption, resulting from irregular patterns of light–dark, sleep–activity, and eating–fasting (Boivin & Boudreau, 2014; Kosmadopoulos et al., 2020; Lunn et al., 2017). Apart from the nature of work occupations, circadian disruption can also be caused by personal lifestyle, caregiving responsibility, or social jet lag. Emerging evidence indicates that circadian disruptions are associated with an elevated susceptibility to cardiometabolic disorders, potentially leading to the pathogenesis of various cardiomyopathies. Notably, cardiovascular disease remains one of the leading causes of global death. Given the increasing prevalence of people with disrupted circadian rhythm and the importance of cardiac health, there is a critical need to investigate circadian‐modulating interventions with therapeutic potential on cardiac function.

It has long been appreciated that cardiometabolic processes exhibit diurnal variations. Up to 10% of the cardiac transcriptome is regulated by the cardiomyocyte circadian clock, impacting various biological processes such as transcription, signal transduction, cellular constituent turnover, transport, and metabolism (Bray et al., 2008; Young et al., 2014). The heart is a non‐stop “engine” of the whole body. During baseline condition, 60%–80% of cardiac energy metabolism relies on fatty acid *β*‐oxidation (Glatz et al., 2020; Lopaschuk et al., 2010). Neutral lipids, as the main fuel source for the heart, are stored in the lipid droplets where cellular lipid metabolism occurs. Maintaining lipid homeostasis is critical in the heart as sufficient lipid substrates are needed to support non‐stop energy needs; on the other hand, excessive lipid derivatives can accumulate and result in lipotoxicity. Lipid metabolism in the heart fluctuates throughout the day. For example, triglyceride synthesis peaks toward the end of the active period (Tsai et al., 2010), while fatty acid oxidation peaks toward the end of the rest phase (Brewer et al., 2018). Furthermore, the synthesis of lipid‐derived signaling molecules increases during the resting phase to prepare for the upcoming wake period (Durgan et al., 2007). This time‐of‐day‐dependent manner of lipid metabolism enables the heart to anticipate the day‐night differences in energy availability and demand, permitting the heart to respond rapidly to a given stimulus at the appropriate time of the day. Unfortunately, metabolic challenges such as aging, diet‐induced obesity, and aberrant eating and fasting patterns could dampen or abolish the time‐of‐day‐dependent manner of lipid metabolism.

Time‐restricted feeding/eating (TRF/TRE in humans) is emerging as a promising behavioral intervention in which food consumption is confined within a consistent window of 6–12 h each day without overtly attempting to limit energy intake (Manoogian, Chow, et al., 2022). This daily feeding‐fasting cycle is believed to act as an environmental cue to maintain or impose daily rhythms of many biological processes, resulting in pleiotropic health benefits in different tissues for organisms from fly and mice to humans (Che et al., 2021; Dantas Machado et al., 2022; Gill et al., 2015; Hatori et al., 2012; Livelo et al., 2023; Mia et al., 2021; Sutton et al., 2018; Villanueva et al., 2019; Wilson et al., 2020; Ye et al., 2020). The positive effects include improvements in glucoregulation, blood pressure, liver triglycerides, plasma lipids, cardiac function, and gut health (Che et al., 2021; Dantas Machado et al., 2022; Gill et al., 2015; Mia et al., 2021; Sutton et al., 2018; Wilson et al., 2020; Ye et al., 2020). These TRF‐mediated benefits may result from modulation of lipid metabolism, inflammation, oxidative stress, RNA splicing and processing, protein folding and processing, ribosome biogenesis, autophagy, cell cycle regulation, mitochondrial functions, and epigenetic modifications (Deota et al., 2023; Mia et al., 2021; Selvaraji et al., 2022). Our lab has previously demonstrated that TRF (12 h feeding: 12 h fasting) attenuates age‐related cardiac declines in *Drosophila* through upregulation of the TCP‐1 ring complex chaperonin and downregulation of mitochondrial electron transport chain complexes (Gill et al., 2015; Melkani et al., 2017). In addition, genetic ablation of clock genes abolishes TRF effects in the heart (Gill et al., 2015). These findings prompted us to further ask whether and how TRF conveys cardiac benefits in *Drosophila* under environmentally induced circadian disruption.

*Drosophila* heart is an attractive model system for dissecting the interplay between cardiac health and the circadian rhythm system. The *Drosophila* heart displays several developmental and functional similarities to the vertebrate heart with growing evidence of recapitulating cardiac dysfunction of human diseases in fruit flies. High‐speed imaging technologies allow for the characterization of heartbeat parameters including heart period (HP), heart rate (HR), arrhythmia index (AI), diastolic interval (DI), systolic interval (SI), diastolic diameter (DD), systolic diameter (SD), and fractional shortening (FS). Besides, the main core clock components in *Drosophila* are highly conserved including Clock (*Clk*), Cycle (*Cyc*), Period (*Per*), and Timeless (*Tim*). Circadian disruption in *Drosophila* can be induced by exposure to constant light. Light lowers PER‐TIM protein levels and inhibits phosphorylation, which inhibits and delays the normal negative feedback regulation (Marrus et al., 1996). In this study, we subjected wild‐type Canton‐S flies to constant light exposure, which served as a model of circadian disruption. We demonstrated that TRF (10 h feeding: 14 h fasting) maintained cardiac performance in both 12 h light: 12 h dark (LD), and constant light (LL) groups. Furthermore, TRF maintained myofibril organization and regulated lipid accumulation in the heart. From transcriptome analysis, we identified that *Dgat2* exhibited a diurnal expression pattern and peaked during the feeding phase under TRF. While heart‐specific constitutive suppression of *Dgat2* led to a higher arrhythmia index (AI), overexpression of human *Dgat2* (h*Dgat2*) resulted in reduced AI. Strikingly, overexpression of hDgat2 from ZT0 to ZT10 led to improvements in arrhythmia index, systolic interval, and fractional shortening. Thus, our results suggest that TRF maintains cardiac health in a *Drosophila* model of circadian disruption, partially through modulation of *Dgat2* expression.

Overall, TRF emerges as a promising intervention, aligning eating patterns with circadian rhythms, thereby improving various health markers, including cardiac function. In a *Drosophila* model, TRF mitigated circadian disruption‐induced cardiac declines, highlighting its potential therapeutic role. This effect is partly mediated by the modulation of *Dgat2* expression, suggesting a molecular mechanism for TRF's cardiac benefits amidst circadian disruption. Understanding these findings is pivotal for developing interventions to mitigate circadian disruption‐related cardiac diseases in humans.

## 2 RESULTS

### 2.1 TRF maintains cardiac health under aging and circadian rhythm disruption

**FIGURE 1:**
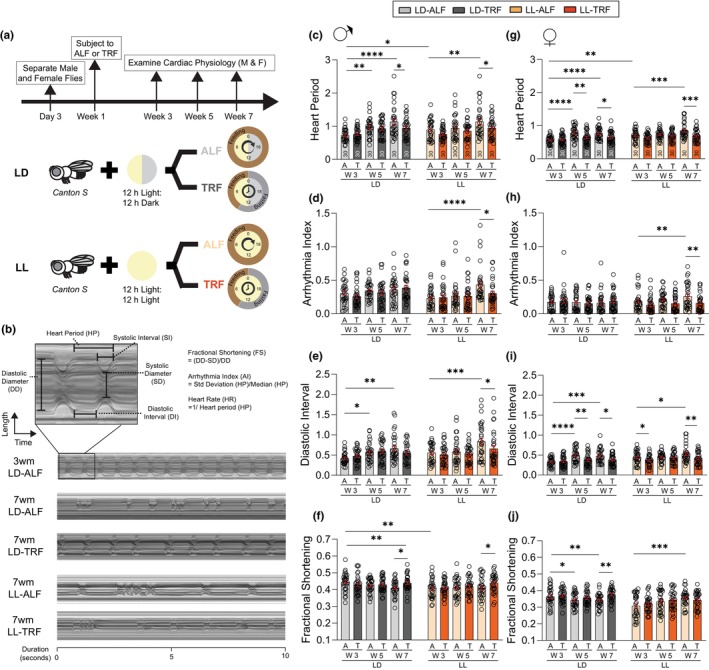
TRF maintains cardiac health under aging and circadian rhythm disruption. (a) Schematic diagram depicting the experimental setup. 1‐week‐old wild‐type Canton S flies were subjected to either 12 h light: 12‐h dark cycle (LD) or constant light (LL). Flies were further subjected to either ALF or TRF. In ALF, flies have unrestricted food access for 24 h. In TRF, flies only have food access from ZT0–10. Cardiac performances were examined at weeks 3, 5, and 7 for both male and female flies. (b) Example of 10 s M‐mode (mechanical mode) traces generated from heart videos. Cardiac parameters calculated from M‐mode include heart period (HP), heart rate (HR), arrhythmia index (AI), diastolic index (DI), systolic index (SI), diastolic diameter (DD), systolic diameter (SD), and fractional shortening (FS). (c, g) HP, (d, h) AI, (e, i) DI, and (f, j) FS were measured from hearts of 3‐, 5‐, and 7‐week‐old LD and LL male and female flies under ALF and TRF. *N* = 30. Mean ± SEM. Three‐way ANOVA with Fisher's LSD tests. *p < 0.05, **p < 0.01, and ***p < 0.001.

To test the impact of TRF on the cardiac performance of flies with disrupted circadian rhythm, we subjected 1‐week‐old wild‐type *Drosophila* (Canton S) adults to either 12 h light: 12‐h dark cycle (LD group) or constant light (LL group). LD and LL groups were then assigned to either ad‐libitum feeding (ALF), where flies have no restriction on food access, or TRF, where food is exclusively available during ZT0–10. Both male and female fly hearts were analyzed to determine any potential sex‐specific impacts of circadian disruption and TRF (Figure 1a). The experiment was repeated with three independent fly cohorts. Flies were dissected under artificial hemolymph, and the heart region at the abdominal segment 2 of the ex vivo contracting hearts was examined by high‐speed video imaging at ZT4–8 in weeks 3, 5, and 7. M‐mode traces were generated to display the dynamics of heart contraction (Figure 1b).

At 3 weeks of age, the cardiac performance of both LD‐ALF and LD‐TRF male hearts was indistinguishable with comparable HP, HR, AI, DI, SI, DD, SD, and FS (Figure 1c–f and Figure S1a). At 7 weeks of age, LD‐ALF male flies exhibited characteristic age‐dependent changes, with significantly increased HP, SI, DI, and reduced DD and FS (Figure 1c–f and Figure S1a). TRF treatment of LD male flies resulted in attenuation of age‐dependent deterioration, with statistical significance on FS (Figure 1c–f). Similar trends were observed in female flies, with additional statistical significance reached from TRF treatment on HP and DI (Figure 1g–j and Figure S1b).

After 2 weeks of constant light exposure, 3‐week‐old LL‐ALF male flies showed significantly increased HP and reduced FS (Figure 1c,f). Continuing light exposure till week 7 further led to significantly increased HP, AI, DI, SI, and reduced DD in 7‐week‐old LL‐ALF flies compared to 3‐week‐old LL‐ALF counterparts (Figure 1c–f and Figure S1a–d). Notably, 7‐week‐old LL‐TRF flies exhibited significant improvements in HP, AI, DI, and SI (Figure 1c–f and Figure S1a–d). Similar results were observed in female flies (Figure 1g–j and Figure S1e–h). Interestingly, age‐dependent reduction in FS was not seen in 7‐week‐old LL‐ALF male flies (Figure 1f), but there was a significant increase in FS in 7‐week‐old LL‐ALF female flies compared to its 3‐week‐old counterparts (Figure 1j). This might be a sex‐specific response to constant light exposure. While TRF further increased FS in 7‐week‐old LL male flies (Figure 1f), no changes were seen in the female counterparts (Figure 1j).

### 2.2 TRF maintains myofibril organization and regulates lipid accumulation

**FIGURE 2:**
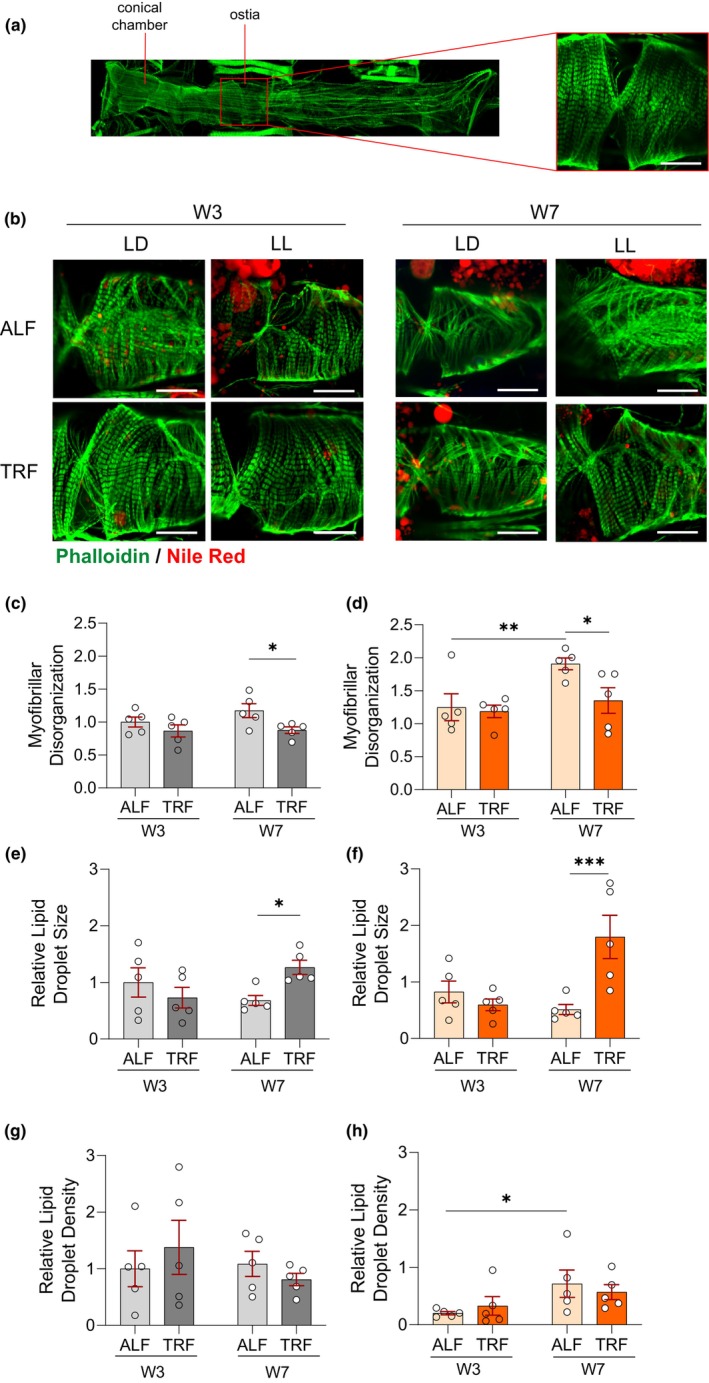
TRF maintains myofibril organization and regulates lipid accumulation. (a) Representative confocal stacks of 1‐week‐old male fly heart (anterior to left) stained with phalloidin for F‐actin‐containing myofibrils. The heart region at abdominal segment 2 is outlined in red revealing details of myofibrillar organization. (b) Representative confocal stacks of hearts from 3‐ and 7‐week‐old LD and LL male flies under ALF and TRF. F‐actin‐containing myofibrils are stained with phalloidin (green) and lipid droplets are stained with Nile Red (red). The scale bar is 25 μm. (c, d) Myofibrillar disorganization of 3‐ and 7‐week‐old LD and LL male flies under ALF and TRF were quantified using Voronoi's diagram. *N* = 5. Mean ± SEM. Two‐way ANOVA with Fisher's LSD test. (e–h) Lipid quantification (relative lipid droplet size (e–f) and density (g–h)). *N* = 5. Mean ± SEM. Two‐way ANOVA with Fisher's LSD test. *p < 0.05, **p < 0.01, and ***p < 0.001.

To investigate whether TRF affects the structural integrity of *Drosophila* hearts, we utilized phalloidin to visualize the F‐actin organization within the myofibrils from the circumferential fibers. Hearts from 3‐ and 7‐week‐old LD and LL male flies under ALF and TRF were collected and fixed at ZT4–8. Myofibril organization was assessed at the same regions where cardiac performance measurements were performed (Figure 2a) and then analyzed using Voronoi's diagram (Selma‐Soriano et al., 2021) (Supplementary 2a, b). In a young and healthy fly heart, myofibrils are paralleled and densely packed (Figure 2a). However, in both LD and LL groups, myofibrils from 7‐week‐old ALF flies exhibited a disorganized fashion, with statistical significance shown in the LL group. Interestingly, myofibril organization from 7‐week‐old TRF flies showed significant improvements compared to their respective ALF counterparts (Figure 2b–d). Consistent with the functional data obtained from beating hearts, cardiac diameter was more restricted in the fixed tissue from 7‐week‐old LD‐ALF hearts compared to its 3‐week‐old counterpart (Figure 2b). These differences in myofibril organization from ALF and TRF hearts may contribute to the differences in cardiac contractibility. We also examined lipid droplets in the *Drosophila* heart using Nile Red staining. Only circular lipid droplets within the myofibril areas were counted during quantification. While there was a trend of reduction in relative lipid droplet size under TRF compared to ALF in 3‐week‐old hearts in both LD and LL flies, interestingly, we observed a significant increase of relative lipid size in 7‐week‐old TRF hearts versus its ALF counterparts (Figure 2e,f). A trend of fewer lipid droplets was observed in 7‐week‐old TRF hearts compared to their age‐matched counterparts but did not reach the level of significance (Figure 2g,h). These results suggested that TRF regulates lipid accumulation in the heart in both LD and LL conditions.

### 2.3 TRF regulates the expression of genes associated with triglyceride metabolism and induces diurnal expression of *Dgat2* in the hearts of flies under constant light

**FIGURE 3:**
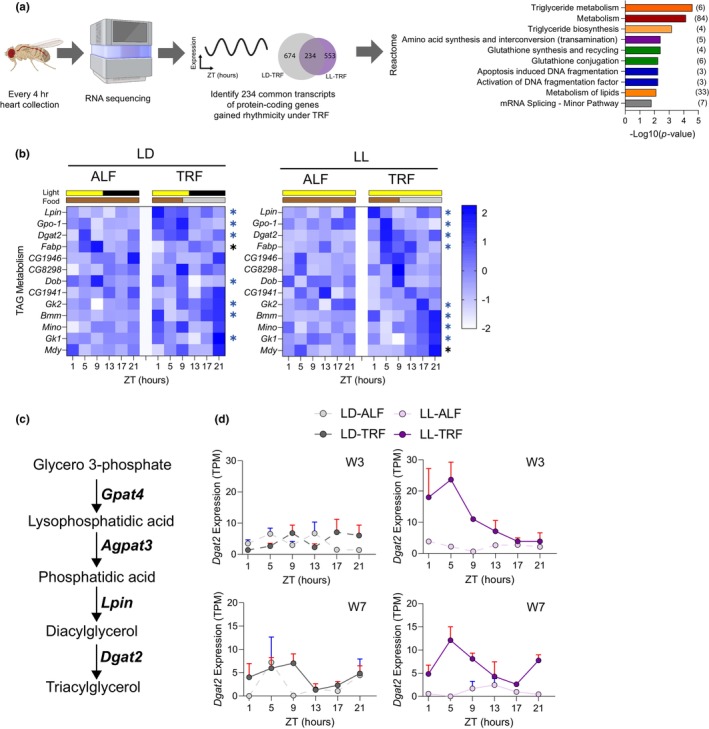
TRF regulates the expression of genes associated with triglyceride metabolism and induces diurnal expression of *Dgat2* when flies are under constant light. (a) Schematic diagram depicting the process of *Drosophila* heart collection, RNA sequencing, rhythmicity analysis, identification of rhythmic transcripts of protein‐coding genes under TRF, and Reactome pathway analysis of 234 overlapped genes that gained rhythmicity under TRF in LD and LL groups. Bar charts represent the −log10 (*p‐*value) of each enriched pathway. Related pathways are colored similarly. The number of genes identified in each pathway is shown in parentheses. (b) Heatmap representation of the temporal expression of genes associated with triglyceride metabolism from 7‐week‐old LD and LL male flies under ALF and TRF. Blue asterisks indicate genes that were unrhythmic under ALF but gained rhythmicity under TRF. Black asterisks indicate genes that were rhythmic under both ALF and TRF with higher amplitude under TRF than ALF. (c) Schematic representation of de novo triacylglycerol synthesis where *Dgat2* catabolizes the last reaction. Metabolite intermediates and important genes (italic bold) encoding enzymes are listed. (d) Expression level of gene *Dgat2* in 3‐week‐ and 7‐week‐old LD and LL male fly hearts under ALF and TRF. *N* = 6 time points. Rhythmic expressions are presented as a solid line, otherwise, a dashed line. The rhythmicity threshold is TPM at all‐time points >0, a TPM maximum/minimum fold‐change ≥1.5, and an Empirical_JTK Benjamini–Hochberg corrected *p* ≤ 0.05.

To gain insight into the potential molecular mechanisms by which TRF protects against circadian disruption‐induced functional changes in hearts, we examined the temporal cardiac transcriptomes from LL and LD flies under ALF and TRF. Fly hearts were collected every 4 h over 24 h from 3‐ and 7‐week‐old male flies (Figure 3a). We performed RNA sequencing and identified genes with a 24‐h cycling pattern of expression using empirical JTK_CYCLE (Hutchison et al., 2015). There were 1642, 1205, 1254, and 1739 rhythmic transcripts of protein‐coding genes in 3‐week‐old LD‐ALF, LD‐TRF, LL‐ALF, and LL‐TRF fly hearts, while 1282, 1579, 1240, and 1309 rhythmic transcripts were identified in 7‐week‐old fly hearts, respectively (Figure S3a).

Expression of core clock genes *Clk* and *Tim* under ALF and TRF was examined (Figure S3b), as *Per* expression was low (TPM <2) and *Cyc* mRNA does not undergo cycling (Akimoto et al., 2005). In general, both *Clk* and *Tim* expression levels oscillate throughout the day with *Clk* peaking within ZT0–12 and *Tim* peaking within ZT12–24. In the LD group, the expression of *Clk* and *Tim* was rhythmic and had similar patterns under ALF and TRF. In the LL group, however, *Tim's* expression levels peaked at ZT5 in 3‐week‐old LL‐ALF hearts but shifted to ZT21 under TRF. On the other hand, *Clk* expression levels peaked at ZT13 in 7‐week‐old LL‐ALF hearts but shifted to ZT9 under TRF. These data not only support that the misalignment of the cardiac clock was induced by constant light but also demonstrated that TRF restored the proper oscillation of *Clk* and *Tim* expression in the heart with environmentally induced circadian disruption.

To investigate the potential pathways modulated by TRF, reactome pathway analysis was performed with 234 overlapped transcripts that gained rhythmicity under TRF in 7‐week‐old LD and LL groups (Figure 3a and Table S1). The common rhythmic genes were enriched in lipid metabolism, amino acid metabolism, glutathione metabolism, DNA fragmentation, and mRNA splicing (Figure 3a). Lipid metabolism in the heart has been previously reported to be regulated by TRF (Mia et al., 2021). Given that TRF modulated lipid content in both LD and LL groups (Figure 2), we further examined the expression patterns of genes related to triglyceride metabolism. As shown in Figure 3b, 7 genes gained rhythmicity under TRF in 7‐week‐old LD and LL flies, respectively, with *Bmm*, *Dgat2*, *Gk1*, *Gk2*, *Gpo‐1*, and *Lpin* in common. Notably, almost all genes associated with triglyceride metabolism oscillated with higher amplitude under TRF (see Table S2 for TPM).

*Dgat2* stood out from genes involved in triglyceride metabolism, as *Dgat2* was identified as playing important roles in TRF‐mediated benefits in muscle from our recent study (Livelo et al., 2023). *DGAT2* is a rate‐limiting enzyme catalyzing the final step in de novo triglyceride synthesis (Figure 3c). While aging and circadian disruption led to a trend of decreased *Dgat2* expression, TRF increased overall *Dgat2* expression (mesor) and reached statistical significance in the LL group. We then further examined *Dgat2* expression at different time points (Figure 3d). In the LD condition, TRF seemed to induce a 4‐h phase shift of *Dgat2* expression compared to ALF. Strikingly, TRF upregulated *Dgat2* expression during the feeding time but not during the fasting period (Figure 3d). This TRF‐induced feeding time‐specific enhancement of *Dgat2* expression may potentially contribute to the TRF‐mediated cardiac benefits.

### 2.4 Cardiac‐specific manipulation of *Dgat2* affects cardiac arrhythmia and lipid accumulation

**FIGURE 4:**
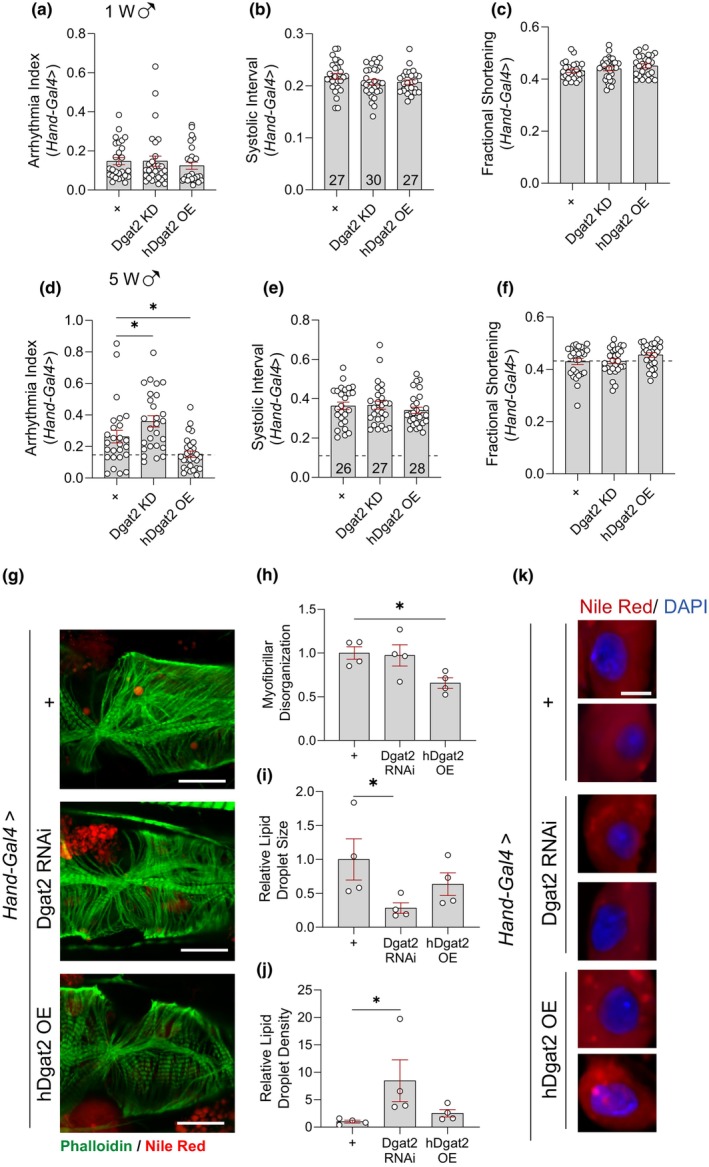
Cardiac‐specific manipulation of *Dgat2* affects arrhythmia index and lipid accumulation. (a–c) AI (a), SI (b), and FS (c) of 1‐week‐old male flies upon suppression of *Dgat2* or overexpression of h*Dgat2*. *N* = 27, 30, and 27, respectively. Mean ± SEM. One‐way ANOVA with Fisher's LSD test. (d–f) AI (d), SI (e) and FS (f) of 5‐week‐old male flies upon suppression of *Dgat2* or overexpression of h*Dgat2*. *N* = 26, 27, and 28, respectively. Mean ± SEM. One‐way ANOVA with Fisher's LSD test. (g) Representative confocal stacks of hearts from 5‐week‐old male flies upon suppression of *Dgat2* or overexpression of h*Dgat2*. F‐actin containing myofibrils are stained with phalloidin and lipid droplets are stained with Nile Red. The scale bar is 25 μm. (h) Myofibrillar disorganization of 5‐week‐old male flies upon suppression of *Dgat2* or overexpression of *hDgat2* was quantified using Voronoi's diagram. (i–j) Lipid quantification (relative lipid droplet size (i) and density (j)). *N* = 4. Mean ± SEM. One‐way ANOVA with Fisher's LSD test. (k) Representative confocal images of myocardiocytes with lipid droplets stained with Nile Red and nucleus stained with DAPI. *p < 0.05, **p < 0.01, and ***p < 0.001.

To understand the role of *Dgat2* in *Drosophila* hearts, we performed knockdown (KD) using UAS‐RNA interference (RNAi) of *Dgat2* and overexpression (OE) of human *Dgat2* (h*Dgat2*) with a heart‐specific driver (*Hand‐Gal4*) in male flies. Gene KD efficiency was assessed using qRT‐PCR (Figure S4a). No significant changes were observed in a 1‐week‐old heart upon *Dgat2* KD and h*Dgat2* OE flies indicating that modulations of *Dgat2* do not cause any heart developmental defects (Figure 4a–c and Figure S4b). To evaluate the effects of *Dgat2* modulations without further age‐associated deteriorations, we assessed the cardiac functions of 5‐week‐old male flies. Interestingly, increased AI was detected in *Dgat2* KD flies (Figure 4d–f and Figure S4c), while a reduction in AI was observed from h*Dgat2* OE flies compared to the age‐matched control (Figure 4a–f). Increased AI was detected in 5‐week‐old *Dgat2* KD female flies, which is similar to what we have observed in male flies. Although decreased AI was also observed in 5‐week‐old h*Dgat2* OE female flies, it does not reach a statistical significance level (Figure S4d).

We also assessed the myofibril organization using phalloidin staining. While heart‐specific suppression of *Dgat2* has similar myofibril organization, flies with heart‐specific h*Dgat2* overexpression showed improvements in the structure compared to the control (Figure 4g,h). Previous studies indicate that *Dgat2* plays an important role in lipid droplet formation and expansion; therefore, we performed Nile Red staining to examine whether manipulation of *Dgat2* expression affects lipid accumulation in the heart. Interestingly, suppression of *Dgat2* led to a smaller size but higher density of lipid droplets compared to the control (Figure 4i,j), while overexpression of h*Dgat2* exhibited more defined lipid droplets within the cardiomyocytes (Figure 4k).

### 2.5 Diurnal expression of h*Dgat2* improves cardiac performance under circadian disruption

**FIGURE 5:**
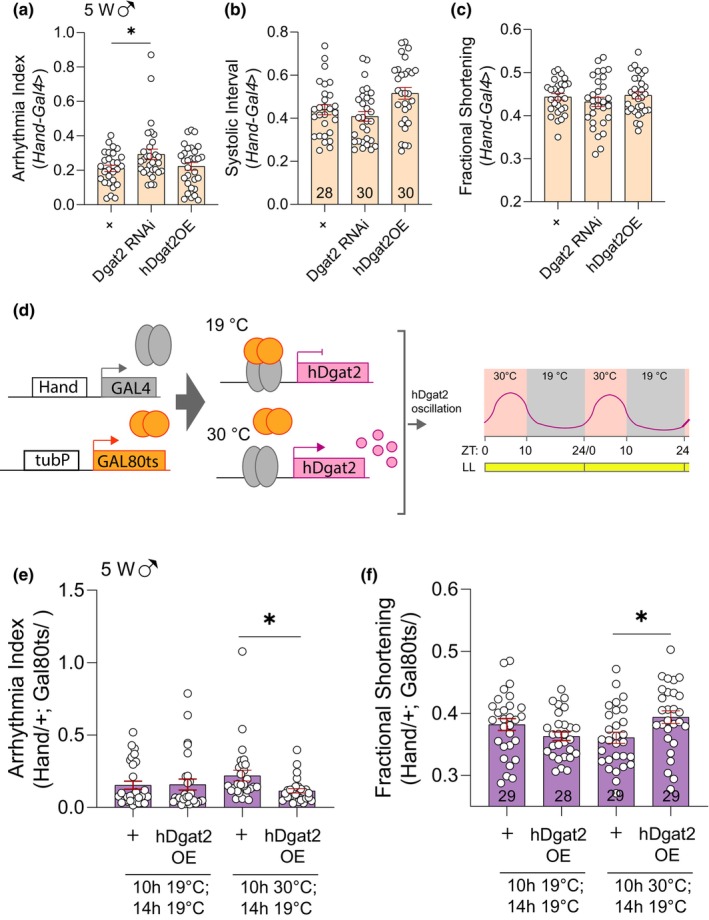
Diurnal expression of h*Dgat2* improves cardiac performance under circadian disruption. (a–c) AI (a), SI (b), and FS (c) of 5‐week‐old male flies upon suppression of *Dgat2* or overexpression of h*Dgat2* under constant light. *N* = 28, 30, and 30, respectively. Mean ± SEM. One‐way ANOVA with Fisher's LSD test. (d) Schematic diagram depicting diurnal overexpression of h*Dgat2* using temperature‐sensitive Gal80ts. Flies were housed at 30°C at ZT0–10 when Gal80ts were released from Gal4 and h*Dgat2* overexpression. Then flies were housed at 19°C at ZT10–24 when Gal80ts bound to Gal4 and h*Dgat2* were not expressed. (e–f) AI (e) and FS (f) of 5‐week‐old male flies upon diurnal overexpression of h*Dgat2* under constant light. Control flies were housed at 19°C at all times. *N* = 29. Mean ± SEM. Two‐sided unpaired *t*‐test. *p < 0.05, **p < 0.01, and ***p < 0.001.

To confirm the role of *Dgat2* in cardiac function, we subjected flies with *Dgat2* KD or h*Dgat2* OE to constant light and examined their heart performance in week 5. However, we did not observe further cardiac deterioration upon *Dgat2* KD under LL (Figure 5a–c and Figure S5a). Given that *Dgat2* expression was elevated only during the feeding period under TRF, we tested whether diurnal induction of h*Dgat2* can improve cardiac performance using Gal80ts when flies were exposed to constant light. Gal80ts is a temperature‐sensitive suppressor of Gal4. Gal80ts is active at 19°C, but not at 30°C. Therefore, by inducing a daily temperature cycle (30°C at ZT0–10; 19°C at ZT10–24), we induced diurnal oscillation of h*Dgat2* expression levels (Figure 5d). First, we examined the heart parameters of the transgenic male flies at 19°C, where hDgat2 was present but not expressed (Figure 5e,f and Figure S5b). HP, HR, AI, DI, SI, and FS were comparable between the hearts of UAS‐hDgat2 flies and the control, except DD and SD. Remarkably, diurnal overexpression of h*Dgat2* improved AI, SI, and FS in 5‐week‐old male flies under constant light when compared with the control (Figure 5e,f and Figure S5c). Similarly, improved SI and FS were also observed in the female flies (Supplementary 5d). These results supported the idea that diurnal upregulation of *Dgat2* expression level induced by TRF may partially contribute to the TRF‐mediated benefits on heart performance.

## 3 DISCUSSION

Currently, several studies have shown that TRF is beneficial to cardiac health; however, whether and how TRF affects heart function in the organism with environmental‐induced circadian disruption is still understudied. Furthermore, although candidate genes or pathways have been identified to be regulated by TRF using multi‐omics platforms, few studies perform functional validations on the contribution of the candidate genes to TRF‐mediated benefits. Herein, first we report that TRF maintains cardiac performance in a *Drosophila* model of circadian disruption induced by constant light, which is accompanied by improvement in myofibril organization and modulation of lipid accumulation. Time‐series transcriptome analysis indicated that TRF‐induced modulation of genes associated with triglyceride metabolism. Particularly, TRF leads to elevation of *Dgat2* expression during the feeding time. Furthermore, *Dgat2* KD led to an increase in AI, while overexpression of h*Dgat2* reduced AI, improved myofibril organization, and increased lipid accumulation. More interestingly, diurnal overexpression of h*Dgat2*, mediated by the temperature‐sensitive driver, improved cardiac parameters including AI, SI, and FS. Thus, our study highlighted the effectiveness of TRF in the heart under environmentally induced circadian disruption and the possibility of developing optimized therapeutic strategies targeting *Dgat2* to improve cardiac health.

Circadian disruption is associated with an increasing risk of cardiometabolic disorders in humans. Indeed, genetic disruption of the murine cardiomyocyte circadian clock abolishes 24‐h rhythms in cardiac metabolism as well as the development of severe cardiomyopathy (Durgan et al., 2011; McGinnis et al., 2017; Tsai et al., 2010). As a growing number of individuals experiencing circadian disturbances, there is an increasing need to explore interventions that can restore circadian rhythms and potentially benefit cardiac health. TRF is an attractive approach as its daily cycle of feeding and fasting has shown its effectiveness in enhancing rhythmicity and synchronization of expression of genes associated with major metabolic pathways (Deota et al., 2023). In addition, the pleiotropic benefits of TRF have been demonstrated in multiple tissues in different animal models and human populations (Che et al., 2021; Gill et al., 2015; Hatori et al., 2012; Livelo et al., 2023; Mia et al., 2021; Sutton et al., 2018; Villanueva et al., 2019). A previous study has shown that TRF‐mediated cardiac benefits were abrogated in *Drosophila* when lacking functional core circadian clock components (Gill et al., 2015). However, it is important to note that genetically induced clock dysfunction is different from environmental‐induced circadian disruption, while the latter would be a better mimic of circadian misalignment caused by a shift in work or lifestyle, which is more commonly seen in humans. Therefore, it is important to understand whether and how TRF is beneficial for heart health under circadian disruption induced by environmental factors. Indeed, a recent human study has examined the effects of TRE on firefighters and showed the feasibility of applying TRE to people with abnormal sleep–wake patterns and the benefits of TRE on cardiometabolic health measures (Manoogian, Zadourian, et al., 2022).

In this study, we first recapitulated that TRF attenuates the age‐related declines in LD flies. Although the trends of aging‐induced deteriorations and TRF‐mediated benefits were consistent with the previous study conducted by our lab (Gill et al., 2015), most of the alterations in cardiac parameters were not statistically significant, which may be due to the usages of different *Drosophila* strains (Canton‐S and Oregon‐R) and TRF regimens (10 h feeding: 14 h fasting and 12 h feeding: 12 h fasting) between the current and previous studies. In the *Drosophila* model of circadian disruption induced by constant light (LL group), we demonstrated that TRF reduced HP, AI, DI, and SI, more importantly, in both male and female flies. Interestingly, potential sex‐specific responses to TRF were observed in cardiac contractility (FS) in the LL flies. In LL condition, reduced FS was observed from 3‐week‐old ALF male flies with no further age‐related deterioration in 7‐week‐old male flies, and TRF flies showed improved FS at the age of week 7. Reduced FS was also observed from 3‐week‐old ALF female flies; however, 7‐week‐old ALF female flies showed higher FS, and no significant changes in FS were observed between ALF and TRF female flies. The unexpected increase in FS from LL‐ALF female flies may be compensation for increased HP; however, further investigation will be needed to explain these sex‐specific phenotypes.

Our previous study demonstrated that TRF led to a reduction of abnormal lipid accumulation in the muscle of *Drosophila* obesity models (Villanueva et al., 2019). Surprisingly, but not unexpectedly, lipid droplet size increased in 7‐week‐old fly hearts under TRF in both LD and LL groups. There are two possible explanations for why larger lipid droplets might be beneficial for cardiac function: (1) Lipid droplets may protect cardiomyocytes from lipotoxicity by sequestering toxic lipids, such as diacylglycerols and ceramides. (2) Lipids that are stored in lipid droplets can be used for hydrolysis to provide fatty acids (FAs) for oxidation to meet energy needs. As Nile Red primarily stains neutral lipids, the details on lipid species within lipid droplets under TRF warrant further investigation.

We identified that TRF induced feeding‐time‐specific elevation of *Dgat2* expression in LL flies. *Dgat2* is one of the acyl‐CoA: diacylglycerol O‐acyltransferase enzymes, which esterifies the diacylglycerol with a fatty acid as the final reaction in the de novo triglyceride synthesis. Heart‐specific suppression of *Dgat2* did not display a striking phenotype in *Drosophila* hearts with significant alteration only in AI. That was not surprising as using an inhibitor of DGAT2 in mice had a very modest effect on triglyceride synthesis in the heart (Roe et al., 2018). Instead, co‐inhibition of DGAT1 and DGAT2 in mice showed significantly lower triglyceride content (Roe et al., 2018). Therefore, *Dgat2* is likely functionally redundant with *mdy* (*Dgat1* homolog in fly). However, *mdy* expression was not rhythmic in 7‐week‐old LD‐TRF flies and was elevated at fasting‐/night‐time in 7‐week‐old LL‐TRF flies. On the other hand, heart‐specific constitutive overexpression of h*Dgat2* in the fly only affects AI but no other heart parameters and is accompanied by more defined lipid droplet formation with more organized myofibril patterns. Several studies have shown that overexpression of *Dgat2* leads to an increase in TAG content (Yamazaki et al., 2005) and larger cytosolic LDs (Liang et al., 2004). Although cardiac TAG content was previously considered a marker of lipotoxic cardiomyopathy, TAG itself may not be toxic. While overexpression of DGAT2 has not been studied in the heart, DGAT1 overexpression shows increased cardiac TAG production and protects the heart from lipotoxicity (Liu et al., 2009) and ischemia–reperfusion injury (Kolwicz Jr. et al., 2015). Besides, a study using adipocyte‐derived cell lines showed that *Dgat2* (but not *Dgat1*) promotes glucose‐derived FAs into a rapidly mobilized pool of TAG, which can be quickly hydrolyzed into FA substrates for mitochondrial β‐oxidation (Irshad et al., 2017). Therefore, it is possible that the upregulation of DGAT2 in the fly heart increases non‐toxic TAG production and promotes fatty acid oxidation for energy needed to maintain cardiac health.

*Dgat2* is a direct clock‐controlled gene. In mammals, the promoter of *Dgat2* contains the E‐box consensus sequences, which can be recognized by the BMAL1/CLOCK heterodimer (Young et al., 2014). Indeed, genetic ablation of either CLOCK or BMAL1 in mice leads to chronic repression of *Dgat2* expression (Young et al., 2014). In this study, we show that constant light exposure in flies disturbs the cardiac circadian rhythm and results in a significant reduction of *Dgat2* expression (7‐week‐old LD‐ALF vs. LL‐ALF). However, TRF restores the rhythmicity of *Dgat2* expression with the expression peak at ZT5 and trough at ZT17 (Figure 3d), potentially facilitating triglyceride synthesis during the feeding period. It is noteworthy that time‐of‐day‐dependent fluctuations are also observed for *bmm* (ATGL), a triglyceride lipase, with the expression peak at the end of the fasting period under the TRF regimen (Figure 3b), potentially facilitating lipolysis during the fasting phase. This activation of lipolysis may provide substrates for energetic demands or potentially promote the synthesis of lipid‐derived signaling molecules during fasting. Together, these observations suggested that TRF induced augmentations of rhythmicity on lipid metabolism as well as a temporal partition of triglyceride synthesis and lipolysis in the hearts of circadian‐disrupted flies. This TRF‐mediated restoration of cardiac metabolic flexibility can contribute to the maintenance of cardiac health. It is therefore not surprising that heart‐specific day‐time overexpression of h*Dgat2* led to more profound improvements in cardiac parameters than constitutively overexpression when compared with their corresponding controls.

It is to be noted that circadian clocks can be entrained by many factors other than feeding protocols, for example, external mechanical vibration (Simoni et al., 2014), social interaction (Levine et al., 2002; Mistlberger & Skene, 2004), drug administration (Turek & Losee‐Olson, 1986), or temperature (Zimmerman et al., 1968). Therefore, daily cycles of these stimuli could potentially restore circadian rhythms under circadian‐disrupted conditions. However, their impacts in the context of cardiac health are largely unknown and would be interesting topics to explore.

In summary, our study demonstrated that TRF attenuated the adverse effects of circadian disruption on cardiac function and myofibrillar integrity. TRF modulated lipid accumulation in the fly heart, with increased lipid droplet size in 7‐week‐old flies. Transcriptomic data showed that TRF restored proper oscillation of *Clk* and *Tim* expression and induced diurnal expression of *Dgat2* peaking during the feeding period. Furthermore, overexpression of human *Dgat2* from ZT0–10 improved cardiac function in flies with constant light‐induced circadian disruption. This study highlights the importance of TRF in maintaining heart health during challenging circadian conditions and identifies the diurnal expression of *Dgat2* as a contributor to TRF‐mediated cardiac benefits in *Drosophila*. These findings might provide potential targets for novel therapeutics for cardiac aging, metabolic disorders, and circadian disruptions in humans.

## 4 METHODS

### 4.1 *Drosophila*, diet, and feeding fasting regimens

Wild‐type *Canton‐S* (Bloomington Drosophila Stock Center [BDSC: 225]) was raised with a standard regular diet: agar 11 g/L, active dry yeast 30 g/L, yellow cornmeal 55 g/L, molasses 72 mL/L, 10% nipagen 8 mL/L, and propionic acid 6 mL/L. Flies were housed at 25 °C, 50% humidity in a 12‐h light/12‐h dark (LD) cycle. Canton S adult flies were collected upon eclosion and then separated into male and female flies in groups of 25–30 on day 3. Flies were assigned a feeding and lighting regimen on day 7. For the LD group, flies were kept in a 12‐h light/12‐h dark cycle. For the LL group, flies were kept at constant light (12 h light/12‐h light). ALF and TRF flies were subjected to vials with regular diet at zeitgeber time zero (ZT0) 8 AM (lights on) and were switched to either a regular food vial (for ALF) or a 1.1% agar vial (for TRF) at 6 PM (lights off). Flies were transferred onto fresh media every 3 days. To evaluate the heart‐specific function of *Dgat2*, transgenic stocks were obtained from Bloomington Drosophila Stock Center (BDSC) and Vienna Drosophila Resource Center (VDRC): UAS‐*Dgat2*‐RNAi (VDRC: 107788), control RNAi (VDRC: 60100), UAS‐human DGAT2 (BDSC: 84854), and UAS‐GFP (BDSC: 5431).

### 4.2 Sample collection, RNA extraction, library preparation, and sequencing

At 3 or 7 weeks of age, hearts were collected from 10 male flies every 4 h over 24 h (ZT1, 5, 9, 13, 17, 21). Two biological replicates per time point were collected. Samples were flash‐frozen and homogenized using a pellet pestle. Total RNA was prepared using the RNeasy kit (QIAGEN). The libraries were pooled and sequenced for paired‐end 150‐bp mRNA sequencing on an Illumina Novaseq 6000 at the UCSD IGM Genomics Center. Analysis of the RNA‐seq data was carried out by The Razavi Newman Integrative Genomics and Bioinformatics Core Facility at the Salk Institute. In brief, raw sequencing data were de‐multiplexed and converted into FASTQ files using CASAVA software (v1.8.2). The quality of read sequences was tested using FASTQC http://www.bioinformatics.babraham.ac.uk/projects/fastqc/. Reads that passed QC (define) were mapped to the *Drosophila* genome (dm6) using STAR aligner software (Dobin et al., 2013) (v2.5.3a) using default parameters (allowing up to 10 mismatches and up to nine multi‐mapping locations per read). Transcript per million gene expression levels were quantified across all exons using the top‐expressed isoforms as a proxy for gene expression using HOMER v4.10 (Heinz et al., 2010).

### 4.3 Rhythmic gene analysis

To analyze the temporal dynamic of gene expression, we used empirical JTK_CYCLE with asymmetry search (Hutchison et al., 2015). Periodic patterns were determined using duplicated data, that is, 2 cycles were artificially created from the one actual cycle of experimental data (Villanueva et al., 2019). Transcripts with TPM at all‐time points >0, a maximum/minimum fold‐change ≥1.5, and a Benjamini–Hochberg corrected *p‐*value ≤ 0.05 were considered periodic. Reactome analyses were performed using a WEB‐based Gene set analysis toolkit (WebGestalt). Gene expression heat maps were generated from transformed values following mean centering. Only limited data are shown in this manuscript as we are performing detailed RNA sequencing analyses, which will be published separately.

### 4.4 Cardiac function assay

The flies were dissected under artificial hemolymph and given 15–30 min to equilibrate in fresh, oxygenated artificial hemolymph. The beating hearts were then captured using high‐speed digital camera (Hamamatsu Flash 4 camera) operating at a frame rate of 200 frames per second (fps) for subsequent analysis of heart function (Fink et al., 2009; Gill et al., 2015; Ocorr et al., 2007), including the characterization of heartbeat parameters including heart period (HP), heart rate (HR), arrhythmia index (AI), diastolic interval (DI), systolic interval (SI), diastolic diameter (DD), systolic diameter (SD), and fractional shortening (FS). The dimensions of the heart were measured at the widest point of the second abdominal chamber during both the systole and diastole phases.

### 4.5 Quantification of myofibril organization and lipid accumulation

Dissected hearts were briefly exposed to 10 mM EGTA for relaxing the heart and then fixed with 4% paraformaldehyde in PBS as previously described (Taghli‐Lamallem et al., 2008). Fixed hearts were stained with 1X fluorescence dye 488‐I with Phalloidin conjugate (1 μg/mL) for the F‐actin‐containing myofibrils and Nile red (1 μg/mL) in 1X PBS for the lipid staining. Fluorescence imaging of *Drosophila* heart tubes was carried out using a Nikon A1R Confocal Microscope. Blinded analysis was performed for quantification of myofibril organization using Voronoi's diagrams (Aurenhammer, 1991; Selma‐Soriano et al., 2021). The cardiac F‐actin‐containing myofibrils were outlined using FIJI software and Voronoi's areas were generated (Figure S2). In organized hearts, the areas obtained are similar and have less variance among them. In disorganized hearts, the F‐actin‐containing myofibrils have gaps in between and have more convoluted paths than in organized hearts. Therefore, the polygon areas of disorganized hearts give higher variance values. Quantification of lipid droplets (size and density) was performed using FIJI software. Briefly, the heart area was selected as the region of interest (ROI) using the polygon tool. Threshold function in Fiji software was applied to visualize the organization and perform measurements of lipid droplets.

### 4.6 Real‐time quantitative PCR

Ten fly hearts were collected and flash frozen for each replicate. RNA was extracted using a RNeasy kit (QIAGEN). Quantitative PCR was performed using SsoAdvanced Universal SYBR Green supermix (Bio‐Rad) in a BIO‐RAD CFX Opus Real‐Time PCR System. Expression was normalized with 60S ribosomal protein (RPL11). Primers for qPCR are listed below: *Dgat2*‐F: TGTCCAAGTTGTTGGTGCTC; *Dgat2*‐R: GGCACTCTTCGAATTCTCCA; *Rpl11*‐F: CGATCTGGGCATCAAGTACGA; *Rpl11*‐R: TTGCGCTTCCTGTGGTTCAC. Results are presented as $2^{-\Delta \Delta C_{t}}$ values normalized to the expression of Rpl11 and control samples. All reactions were performed in triplicate.

### 4.7 Statistical analysis

Significance in cardiac function was determined using one‐way ANOVA or two‐way ANOVA with Fisher's LSD test method for multiple comparisons or two‐sided unpaired *t*‐tests. Significance in myofibril organization was performed using one‐way or two‐way ANOVA with Fisher's LSD test method. Lipid droplet size and density differences were performed by one‐way ANOVA with Fisher's LSD test method or two‐sided unpaired *t*‐tests. Significance in qPCR results was determined by Two‐way ANOVA with Fisher's LSD test method or two‐sided unpaired *t*‐test. Bar graphs show Mean ± SEM. All statistical analyses were performed with GraphPad Prism 9.

## AUTHOR CONTRIBUTION

GCM and YG designed the experiments in consultation with SP. YG and HDL prepared sequencing samples. YG analyzed transcriptomic data, performed cardiac functional assays and analysis, acquired and analysis of cytological imaging, and qPCR experiments and analysis with help from FA. YG prepared the paper with GCM's input. All authors provided feedback on the manuscript.

## CONFLICT OF INTEREST STATEMENT

The authors declare no competing interests.

## Supporting information


Figure S1–S5.

Tables S1–S2.

## Data Availability

The data that support the findings of this study are available from the corresponding author upon reasonable request.
