# Supplementary material for: Diurnal expression of Dgat2 induced by time‐restricted feeding maintains cardiac health in the Drosophila model of circadian disruption
Source: Aging Cell. 2024 Apr 14;23(7):e14169. doi: 10.1111/acel.14169 (PMC11258440; doi:10.1111/acel.14169)
Supplement: Supplementary file 1 — Figure S1–S5. [file ACEL-23-e14169-s001.zip › Guo et al_Supporting Information_Aging Cell_ACE-23-0745 (1).pdf]

Diurnal expression of *Dgat2* induced by time-restricted feeding maintains cardiac health in the *Drosophila* model of circadian disruption

Yiming Guo[1], Farah Abou Daya[1], Hiep Dinh Le[2], Satchidananda Panda[2], Girish Melkani[1]*

[1]Department of Pathology, Division of Molecular and Cellular Pathology, Heersink School of Medicine, University of Alabama at Birmingham, Birmingham, AL 35294, USA.
[2]Regulatory Biology Laboratory, Salk Institute for Biological Studies, La Jolla, CA 92037, USA.

[*]Corresponding Address: Department of Pathology, Division of Molecular and Cellular Pathology, Heersink School of Medicine, University of Alabama at Birmingham, Birmingham, AL 35294, USA. Tel.: 1-205-996-0591; Fax: 1-205-934-7447; E-mail: girishmelkani@uabmc.edu (GCM).

Supplementary Data: A Single PDF contains five Figures and Legends.

Supplementary Figure 1

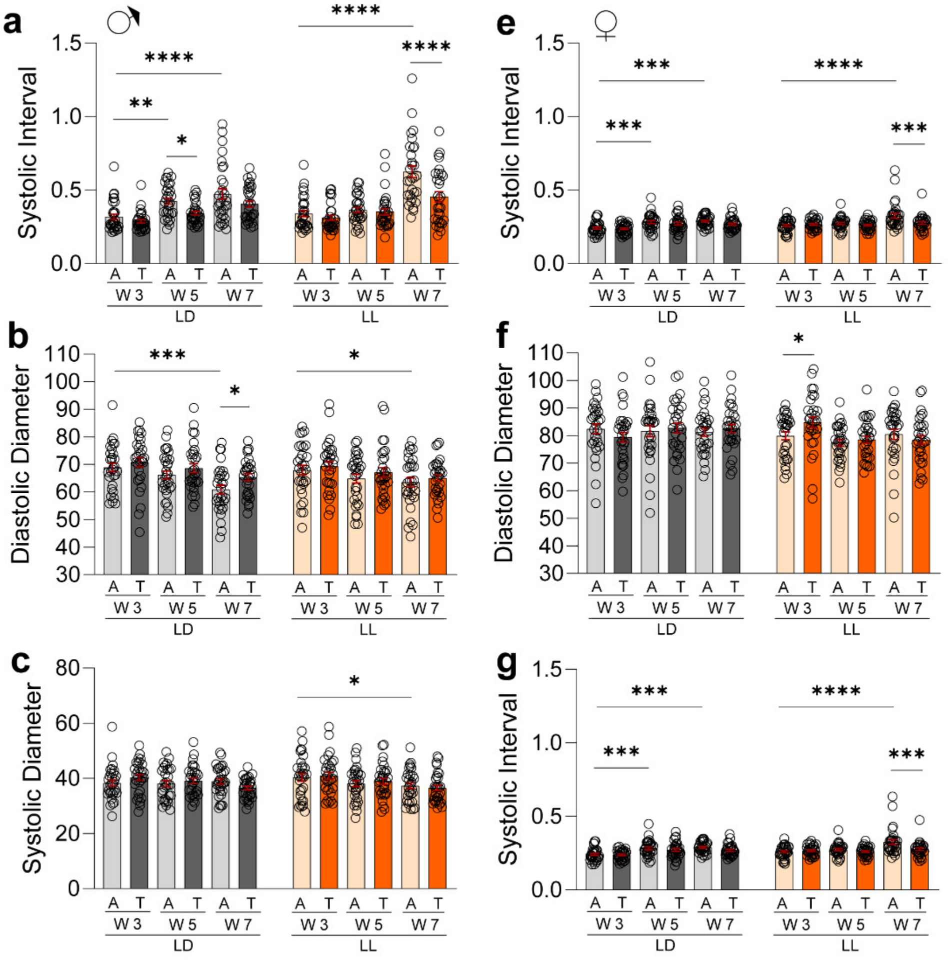

d

♂	Aging		LL		Time-restricted feeding	
	7w LD-ALF vs. 3w LD-ALF	7w LL-ALF vs. 3w LL-ALF	3w LL-ALF vs. 3w LD-ALF	7w LL-ALF vs. 7w LD-ALF	7w LD-TRF vs. 7w LD-ALF	7w LL-TRF vs. 7w LL-ALF
HP	↑↑	↑↑	↑↑	↑↑	↓	↓↓
AI	↑	↑↑	—	↑	—	↓↓
DI	↑↑	↑↑	↑	↑↑	↓	↓↓
SI	↑↑	↑↑	—	↑↑	↓	↓↓
DD	↓↓	↓↓	—	—	↑↑	—
SD	—	↓↓	—	—	—	—
FS	↓↓	—	↓↓	—	↑↑	↑↑

h

♀	Aging		LL		Time-restricted feeding	
	7w LD-ALF vs. 3w LD-ALF	7w LL-ALF vs. 3w LL-ALF	3w LL-ALF vs. 3w LD-ALF	7w LL-ALF vs. 7w LD-ALF	7w LD-TRF vs. 7w LD-ALF	7w LL-TRF vs. 7w LL-ALF
HP	↑↑	↑↑	↑↑	↑	↓↓	↓↓
AI	—	↑↑	—	↑↑	—	↓↓
DI	↑↑	↑↑	↑↑	—	↓↓	↓↓
SI	↑↑	↑↑	↑	↑↑	↓	↓↓
DD	—	—	—	—	—	—
SD	—	↓↓	—	—	—	—
FS	↓↓	↑↑	↓↓	—	↑↑	—

SUPPLEMENTARY FIGURE 1: TRF maintains cardiac function under aging and circadian rhythm disruption.

SI (a, e), DD (b, f), and SD (c, g) were measured from hearts of 3-, 5- and 7-week-old LD and LL male and female flies under ALF and TRF. N=30. Mean ± SEM. Two-way ANOVA with Fisher's LSD tests. *P < 0.05, **P < 0.01, and ***P < 0.001. (i-j) Summary table of cardiac parameters from hearts of 3-week- and 7-week-old LD and LL male (i) and female (j) flies under ALF and TRF. One arrow indicates a trend without reaching statistical significance (P value > 0.05 but ≤ 0.2). Two arrows indicate statistical significance.

Supplementary Figure 2

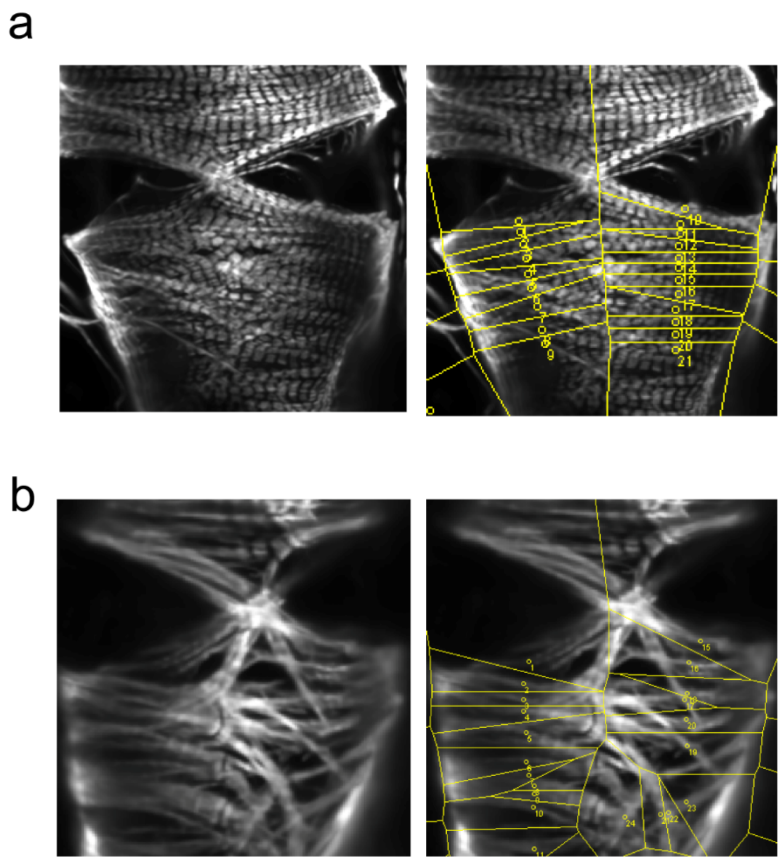

SUPPLEMENTARY FIGURE 2: Quantification of myofibril organization using Voronoi's diagram.

(a-b) Examples of organized (a) and disorganized (b) hearts outlined using Voronoi's diagram.

Supplementary Figure 3

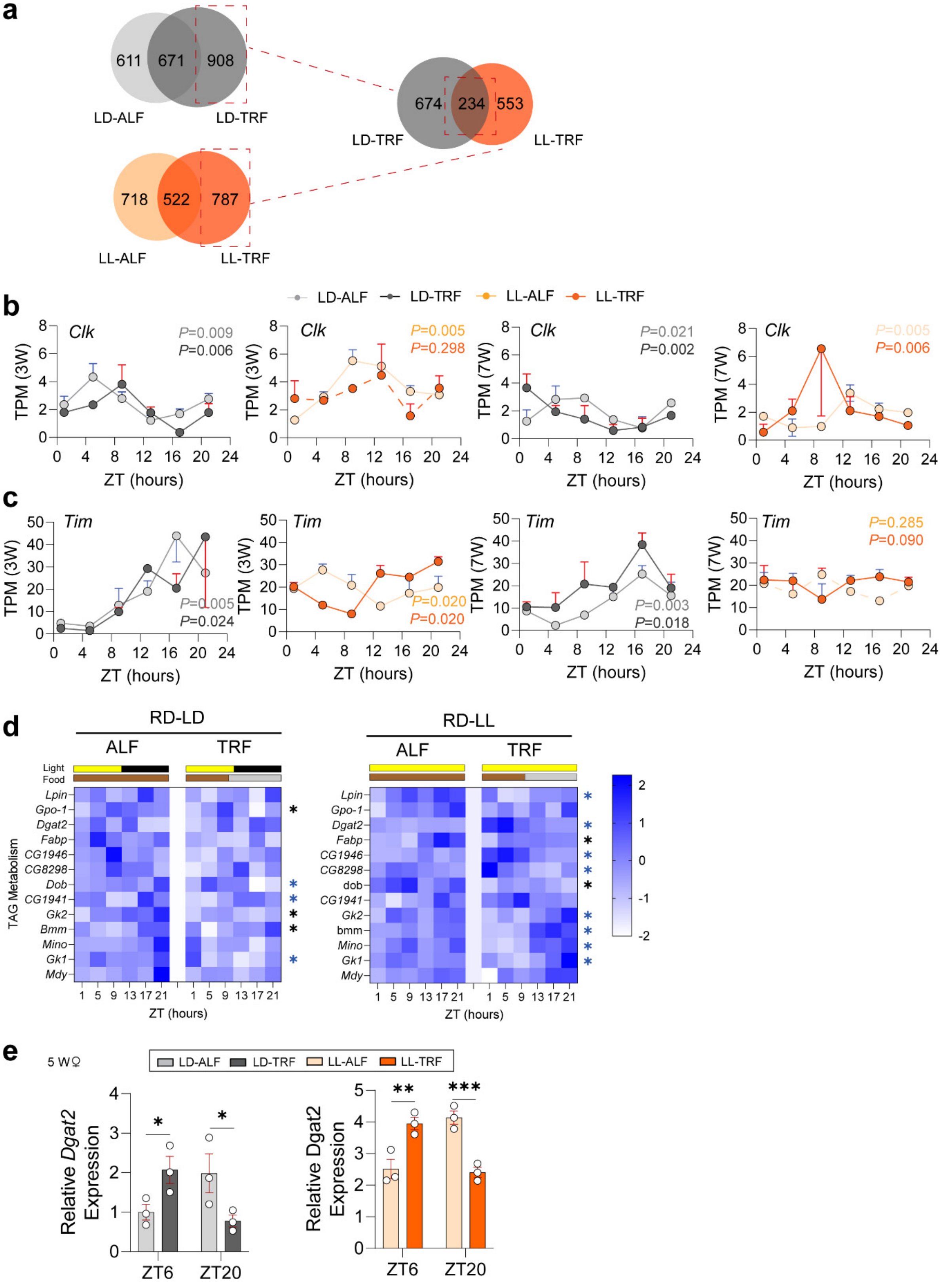

SUPPLEMENTARY FIGURE 3: TRF induces expression rhythmicity of genes associated with triglyceride metabolism and induces diurnal expression of *Dgat2* when flies are under constant light.

(a) Rhythmic cardiac transcripts of protein-coding genes identified under ALF and TRF in LD and LL groups. Transcripts gained rhythmicity under TRF in both LD and LL flies were highlighted in red with 234 transcripts overlapped. (b-c) Expression levels of *Clk* and *Tim* under ALF and TRF in 3-week-old (b) or 7-week-old, Mean ± SEM (c) LD and LL fly hearts. Rhythmic expression are presented as a solid line, otherwise, a dash line. Rhythmicity threshold is TPM at all time points >0, a TPM maximum/minimum fold-change ≥ 1.5 and an Empirical_JTK Benjamini-Hochberg corrected $P \leq 0.05$. (d) Heatmap representation of temporal expression of genes associated with triglyceride metabolism from 3-week-old LD and LL male flies under ALF and TRF. Blue asterisks indicate genes that were unrhythmic under ALF but gained rhythmicity under TRF. Black asterisks indicate genes that were rhythmic under both ALF and TRF with higher amplitude under TRF than ALF. (e) *Dgat2* expression levels from 5-week-old LD and LL female fly hearts at ZT 6 and ZT 20. N=3. Mean ± SEM. Two-way ANOVA with Fisher's LSD tests. *$P < 0.05$, **$P < 0.01$, and ***$P < 0.001$.

Supplementary Figure 4

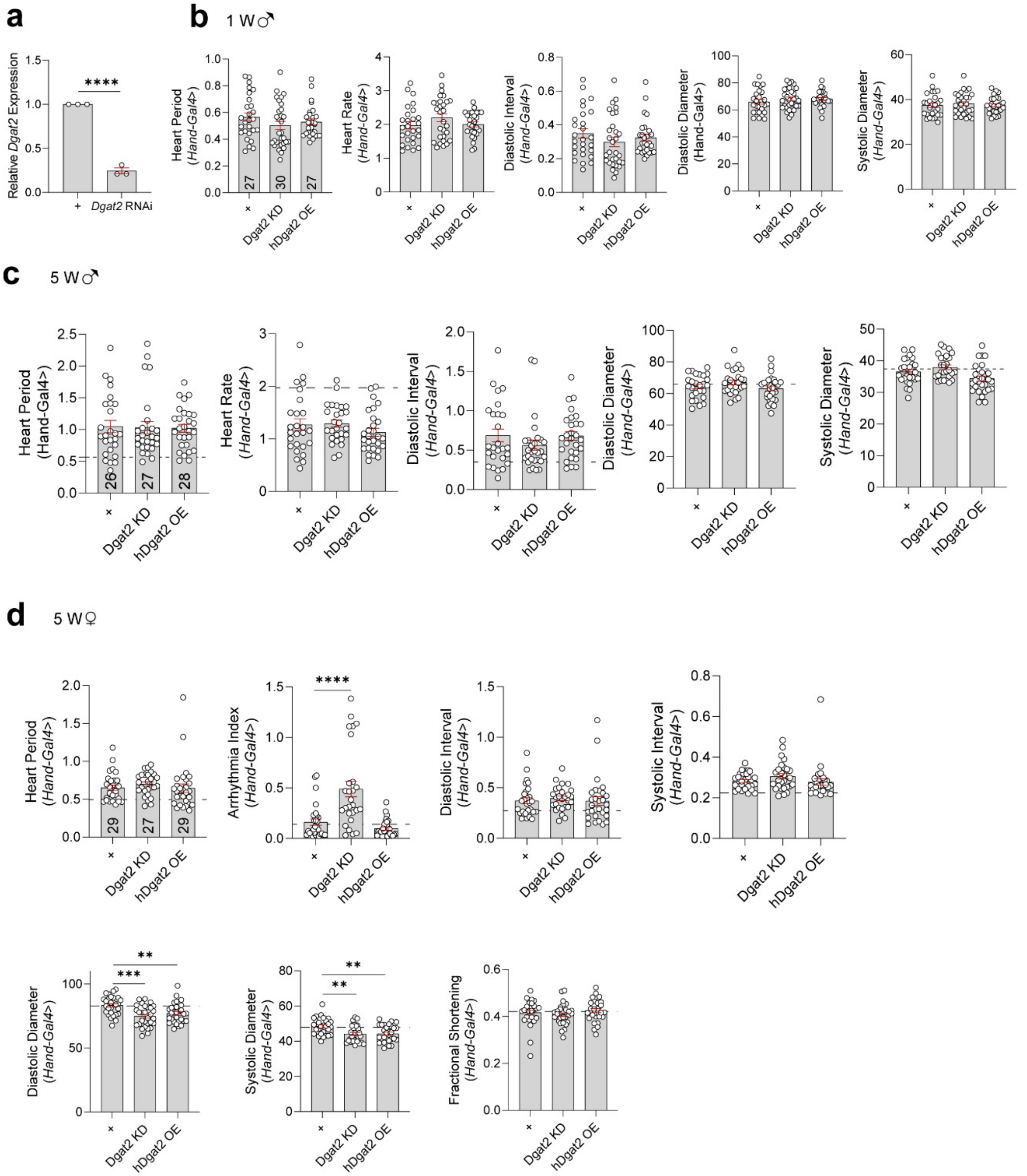

SUPPLEMENTARY FIGURE 4: Cardiac-specific manipulation of *Dgat2* affects arrhythmia index and lipid accumulation.

(a)Relative expression of *Dgat2* in 1-week-old male fly hearts upon *Hand-Gal4* driven *Dgat2* KD were quantified with qRT-PCR. N = 3. Mean ± SEM. Two-sided unpaired t-test. (b-c) Heart parameters of 1-week-old (b) or 5-week-old (c) male flies upon *Dgat2* KD or h*Dgat2* overexpression. (d) 5-week-old female flies upon *Dgat2* KD or h*Dgat2* overexpression. N is indicated. Mean ± SEM. One-way ANOVA with Fisher's LSD tests. *P < 0.05, **P < 0.01, and ***P < 0.001.

Supplementary Figure 5

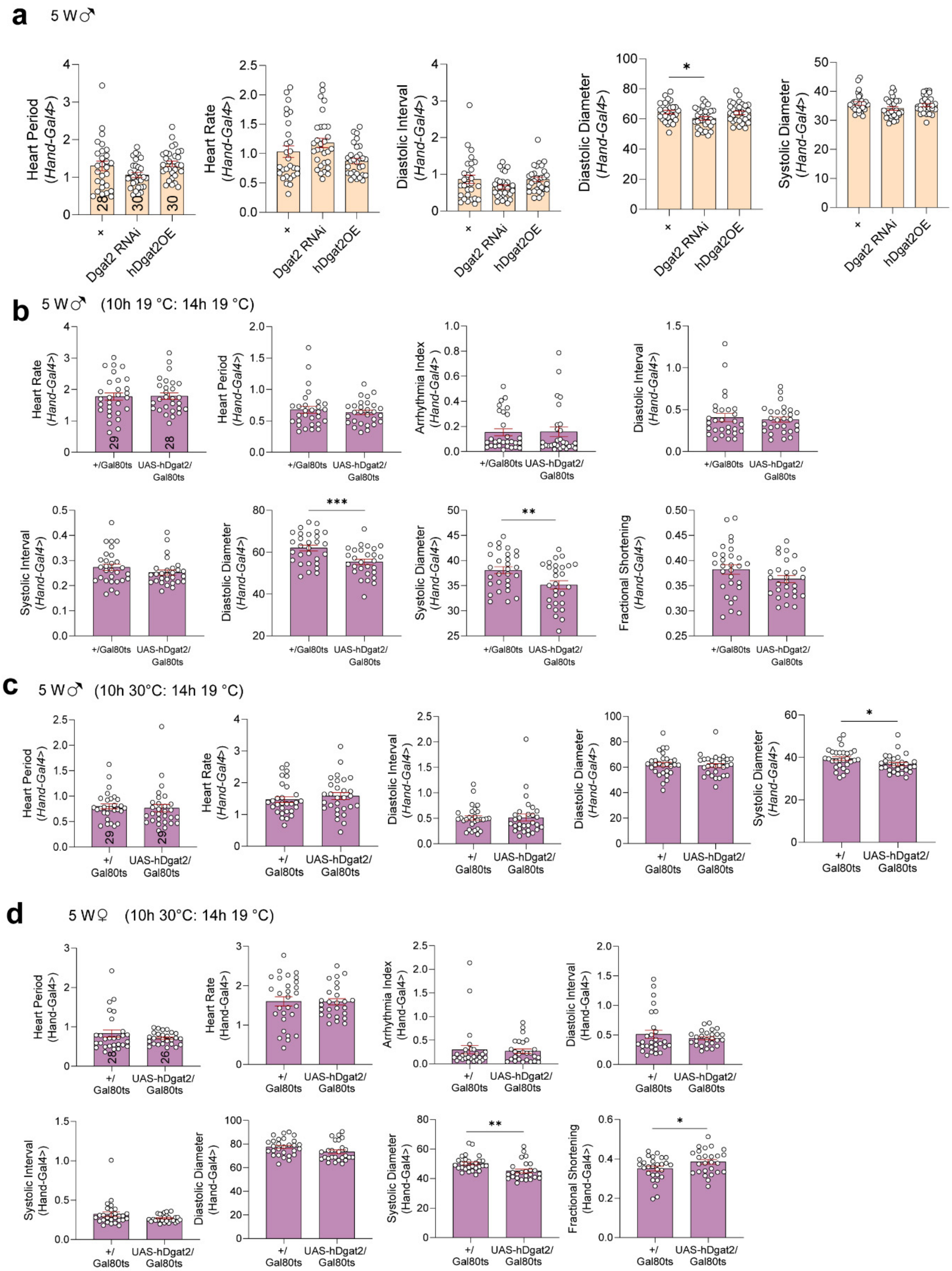

SUPPLEMENTARY FIGURE 5: Diurnal expression of h*Dgat2* improves cardiac performance under circadian disruption.

(a) Heart parameters of 5-week-old male flies upon *Dgat2* KD or h*Dgat2* overexpression under constant light. N is indicated. Mean ± SEM. One-way ANOVA with Fisher's LSD tests. (b) Heart parameters of 5-week-old male flies with indicated genotypes at 19 °C at all times. (c-d) Heart parameters of 5-week-old male (c) and female (d) flies upon diurnal h*Dgat2* overexpression under constant light. N is indicated. Mean ± SEM. Two-sided unpaired t-tests. *P < 0.05, **P < 0.01, and ***P < 0.001.
